# Brain‐Responsive Neurostimulation for the treatment of adults with epilepsy in tuberous sclerosis complex: A case series

**DOI:** 10.1002/epi4.12481

**Published:** 2021-03-13

**Authors:** Danielle S. McDermott, Emily A. Mirro, Kirsten Fetrow, David E. Burdette, Stephanie Chen, Jennifer Hopp, Todd Masel, Emily A. Johnson, Felicia M. K. Elefant, Scheherazade Le, Sanjay E. Patra, Mesha‐Gay Brown, Zulfi Haneef

**Affiliations:** ^1^ University of Colorado Anschutz Medical Campus Aurora CO USA; ^2^ NeuroPace, Inc. Mountain View CA USA; ^3^ Spectrum Health System Grand Rapids MI USA; ^4^ University of Maryland Medical Center Baltimore MD USA; ^5^ University of Texas Medical Branch Galveston TX USA; ^6^ Washington University School of Medicine St. Louis MO USA; ^7^ Stanford University Palo Alto CA USA; ^8^ Baylor College of Medicine Houston TX USA

**Keywords:** refractory epilepsy, responsive neurostimulation, tuberous sclerosis complex

## Abstract

**Objective:**

Tuberous sclerosis complex (TSC) is a genetic disorder primarily characterized by the development of multisystem benign tumors. Epilepsy is the most common neurologic manifestation, affecting 80%‐90% of TSC patients. The diffuse structural brain abnormalities and the multifocal nature of epilepsy in TSC pose diagnostic challenges when evaluating patients for epilepsy surgery.

**Methods:**

We retrospectively reviewed the safety experience and efficacy outcomes of five adult TSC patients who were treated with direct brain‐responsive neurostimulation (RNS System, NeuroPace, Inc).

**Results:**

The average follow‐up duration was 20 months. All five patients were responders (≥50% disabling seizure reduction) at last follow‐up. The median reduction in disabling seizures was 58% at 1 year and 88% at last follow‐up. Three of the five patients experienced some period of seizure freedom ranging from 3 months to over 1 year.

**Significance:**

In this small case series, we report the first safety experience and efficacy outcomes in patients with TSC‐associated drug‐resistant focal epilepsy treated with direct brain‐responsive neurostimulation.

**Key Points**

- TSC patients commonly have comorbid drug‐resistant epilepsy that is difficult to treat with epilepsy surgery.
- Five TSC patients were treated with brain‐responsive neurostimulation (RNS System) for an average of 20 months.
- All five patients experienced >50% seizure frequency reduction at last follow‐up.
- Brain‐responsive neurostimulation was well tolerated and efficacious in this small series of TSC patients with focal drug‐resistant epilepsy.

## 1 INTRODUCTION

Tuberous sclerosis complex (TSC) has a prevalence of 1 in 6000 people.^1^ Epilepsy is the most common neurologic condition associated with TSC and has a prevalence of roughly 80%‐90%.^2^, ^3^ Over 60% of TSC patients with epilepsy are refractory to antiseizure medication (ASM).^4^ The proportion of TSC patients refractory to ASM is twice that of the general epilepsy population.^5^ Infantile spasm is a common seizure type in TSC patients; however, 68% of TSC patients have more than one focal onset seizure type, including focal aware, focal impaired awareness, and focal to bilateral tonic‐clonic seizures.^2^, ^3^, ^4^

Resective or ablative surgery is considered the gold standard therapy for TSC‐associated drug‐resistant epilepsy (TSC‐DRE) when a seizure focus or foci can be clearly identified. Tuber resection has shown efficacy in patients with epileptogenic tubers and has been associated with improved cognitive outcomes when successful.^6^, ^7^, ^8^ However, for many TSC‐DRE patients, focal or tuber resection is not a suitable option. Numerous seizure foci, difficult to treat seizure foci (eg, bilateral or near eloquent cortex), and generation of secondary foci that propagate seizures postoperatively may contribute to unsuccessful resective or ablative procedures.^9^ Additionally, seizure foci are not always associated with tubers. These factors make TSC‐DRE exceedingly difficult to manage. Neuromodulation offers new treatment options for this patient group.

The RNS System (NeuroPace, Inc) delivers direct brain‐responsive neurostimulation to one or two seizure foci in response to abnormal epileptiform activity detected by the system. In addition to the efficacy reported during the clinical trials,^10^ it was also demonstrated that areas of eloquent cortex could be treated without stimulation side effects.^11^ There were no patients with diagnosed TSC in the RNS System clinical trials, perhaps due to the concern that repeated MRI scans would be needed to monitor tuber growth. Here, we report safety experience and efficacy outcomes for five cases of TSC‐DRE treated with the RNS System.

## 2 METHODS

A retrospective chart review was performed on adult patients with TSC‐DRE across five institutions who were treated with the RNS System according to the FDA approved indication for use between November 20, 2016, and October 1, 2019. Each institution received approval from their respective institutional review boards to perform this review. Inclusion criteria were a diagnosis of TSC by diagnostic or genetic testing, and treatment with the RNS System for a minimum of 6 months. All patients underwent surgical localization, and those results guided the location and type of leads implanted.

Demographics, epilepsy histories, RNS System details, serious adverse events and stimulation‐related side effects were collected from the patients’ medical records. Disabling seizure frequencies were collected from the medical record and reported as an average of seizures per month over the prior year. The disabling seizure frequencies per month were collected for the following time‐points: preimplant baseline, 1 year postimplantation and last follow‐up postimplant. Disabling seizures were defined as focal aware (with motor component), focal impaired awareness, or focal to bilateral tonic‐clonic seizures. Changes from baseline disabling seizure frequency were calculated for 1 year and last follow‐up postimplant. Safety was measured through review of serious adverse events. The Clinician Global Impression Scale (CGIS) was obtained from treating physicians to assess patients’ clinical responses to RNS System treatment as of the last follow‐up visit.

## 3 RESULTS

Five adult patients diagnosed with TSC‐DRE and focal epilepsy were treated with the RNS System per the FDA indication for use. Demographics, epilepsy history, and RNS System lead placement can be found in Table 1. The median age of our patients was 35 years (range: 23‐41). No patient had a history of infantile spasms or generalized epilepsy. The results of MRI demonstrated that all patients were found to have cortical tubers in multiple lobes of the brain, and no patient had mesial temporal sclerosis. Other MRI findings can be found in Table 1. All patients underwent intracranial monitoring evaluation. Three of the five patients had the RNS System placed at the same surgery as intracranial monitoring electrode removal.

**FIGURE 1:**
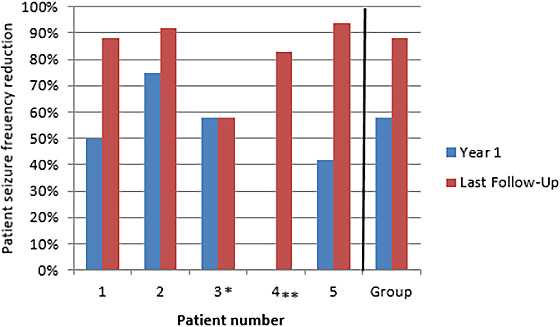
Patient seizure frequency reduction at 1 y and last follow‐up appointment compared with preimplantation baseline

The median baseline seizure frequency was 12 disabling seizures per month (mean: 33.6, range: 2‐88 seizures). The median reduction in disabling seizures was 58% at 1 year (mean: 62%, range: 42%‐95%) and 88% (mean: 83%, range: 58%‐95%) at last follow‐up post–RNS implant. The median follow‐up duration post–RNS implantation was 17 months (range: 9‐33 months). All five patients were responders, defined as a 50% or greater reduction in clinically disabling seizures at last follow‐up. Three of the five patients experienced some period of seizure freedom ranging from less than 3 months to over 1 year. All patients were categorized as “Much Improved” or “Very Much Improved” on the CGIS. The individual reductions in disabling seizures at year one and last follow‐up can be found in Figure 1. One patient (patient 2) was able to reduce their ASMs from two medications to one medication. All other patients remained on the same number of ASMs throughout the period of follow‐up reviewed.

**TABLE 1:** Patient demographics, Epilepsy and TSC history, and RNS lead location

Pt #	Sex	Age	Age diagnosed with Epilepsy	MRI findings	TSC outside of brain	Prior epilepsy surgery	Genetic testing	Neuropsychological assessment	Seizure localization	Baseline Seizure count (per month)	Most recent follow‐up seizure count (per month)	RNS lead placement in relationship to tuber(s)	Rationale for RNS
1	F	41	8 yo	**Tuber** **location**: All lobes **SEGA:** No **Subependymal** **nodules**: No	Kidney‐ angiomyolipoma Skin‐angiofibroma Dental‐pitting of enamel.	No	No	Prominent deficits verbal/language‐verbal memory; visual learning and memory intact.	Left mesial temporal	12	2	**Active:** Subtemporal cortical strips **Connected,** **not** **active:** Cortical strip over tuber	Less Risk to the patient's functionality from a cognitive perspective.
2	M	23	11 yo	**Tuber** **location:** **Multilobar** **including** **one** **large** **left** mesial temporal **SEGA**: Yes **Subependymal** **nodules:** No	Skin‐ Shagreen patch	No	No	Verbal memory, nonverbal memory, expressive language, visuoperceptual abilities all affected	Left lateral temporal	2	0.33	**Active:** Left temporal cortical strips over tuber	Onset thought to be more cortical near language cortex and therefore the more conservative approach
3	M	37	2 d	**Tuber** **location:** Frontal, temporal parietal **SEGA:** No **Subependymal** **nodules:** Yes	No	Partial right frontal lobectomy	Yes, at outside center, positive for TSC per family	IQ 48, deficiencies in all areas	Right frontocentral	60	25	**Active:** Right frontocentral cortical strips over tuber	To preserve the patient's Left hand/arm motor function
4	F	35	14 yo	**Tuber** **location:** All lobes **SEGA:** Yes **Subependymal** **nodules:** Yes **Other:** Linear FLAIR hyperintense bands radiating from periventricular white matter to subcortical region.	No	No	Variant of uncertain significance identified in TSC1, c.362A > T (p. Lys121Met),heterozygous	Left frontotemporal compromise	Left mesial temporal with rapid propagation to neocortical lateral temporal lobe	6	1	**Active:** Left lateral temporal cortical strip and hippocampal depth adjacent to left posterior temporal tuber	Dominant hemisphere and wide network of seizure onset zone on Phase II
5	F	25	14 mo	**Tuber** **location:** Frontal and Parietal **SEGA:** No **Subependymal** **nodules:** Yes	Skin‐angiofibroma	Left frontal and Left parietal	No	Verbal Comprehension abilities (SS = 63) and Perceptual Reasoning abilities (SS = 60)	Bilateral Fronto‐parietal	88	5	**Active:** Left frontal (posterior to previous resection) and centromedian nucleus of left thalamus **Inactive:** Right frontal (posterior to previous resection) and centromedian nucleus of right	Broad bihemispheric field precludes further resection

Abbreviation: SEGA, Subependymal Giant Cell Astrocytoma.

There were no serious adverse events reported in any patient. Stimulation‐related side effects were noted in two patients (patient 4 and 5). Patient 4 had intermittent intense left periorbital pain and muscle twitching time locked with stimulation suggesting meningeal irritation; pain subsided with decreased stimulation parameters. Patient 5 would occasionally report right hand dysesthesias when stimulation was delivered to electrodes placed in the centromedian nucleus of the thalamus. The patient elected to continue with the stimulation as it was not considered bothersome.

## 4 DISCUSSION

This is the first report of TSC‐DRE treated with brain‐responsive neurostimulation. The five patients in our cohort experienced median reductions in clinical seizures of 58% at 1 year and 88% at last follow‐up with the RNS System. The RNS System implantation procedure and responsive stimulation delivery were without complication in this small cohort.

Resective surgery can be successful in TSC patients who have a single seizure focus and/or a MRI lesion that is concordant with the seizure focus. However, TSC patients frequently have more than one seizure focus. Over 40% of TSC patients that undergo resective surgery continue to have seizures.^12^ Two of the patients in our cohort had prior resective surgery with continued seizures. The RNS System can be used in combination with a resective procedure if some tissue can be safely resected but other cortex involved in the seizure‐onset region is eloquent or unresectable. Additionally, the electrographic seizure data gathered by the RNS System can guide future resective surgery.^13^, ^14^

Epilepsy surgery evaluations often uncover regional seizure networks in TSC patients that may involve more than one epileptogenic tuber, the rim of the tuber, and the cortex overlying the tuber.^15^, ^16^ The RNS System offers the flexibility of treating two distinct seizure foci^11^ or larger regional neocortical seizure foci.^17^, ^18^ The neurostimulator can be connected to two depth and/or cortical strip leads at one time and up to four leads (at most two depth leads) can be placed. The additional leads are reserved for future connection to the neurostimulator if desired. The depth leads have two electrode spacing options which allow the targeting of a small structure (such as a tuber) with a compact spacing (four electrodes, 3.5 mm center‐to‐center distance) or a broader area (such as a tuber and its overlying cortex) with a longer spacing (10 mm center‐to‐center distance). In our case series, the RNS System was used successfully to treat patients with dual foci and patients with a single broad region of seizure onset.

Seizure outcomes in patients that have undergone epilepsy surgery for TSC‐DRE decrease over time from 75% seizure freedom at 1 year to 48% at 10 years.^19^ In contrast, the RNS System clinical trials demonstrated an improvement in clinical seizures over time, a median of 44% reduction at 1 year^20^ to 75% at 9 years.^10^ A recent posttrial report of real‐world outcomes reported similar improvements over time but with larger improvements observed earlier in the course of treatment, a median of 67% at 1 year to 82% at 3 or more years.^21^ Our patients experienced reductions similar to that of the published real‐world study, with a median reduction in seizures of 58% at 1 year and a median of 88% in an average follow‐up of 20 months.

The RNS System recently received FDA approval for MRI conditional labeling, allowing patients with a RNS^®^ Neurostimulator model RNS‐320 to undergo a 1.5T full‐body MRI scan under certain conditions. This new labeling makes it possible for patients with TSC to be treated with brain‐responsive neurostimulation while still allowing MRI scans to monitor brain and other organ tuber growth.

### 4.1 Study limitations

This case series is limited by a small sample size and retrospective design. The retrospective nature of a chart review has potential to bias data reporting. It should be noted that while it is fairly common to have generalized seizure types in TSC, the FDA approved indication for use for the RNS System necessitated that our group of patients had focal epilepsy. The safety and efficacy of the RNS System treatment of TSC patients should continue to be evaluated in additional patients.

## 5 CONCLUSION

These data suggest that brain‐responsive neurostimulation with the RNS System can be a safe and appropriate therapeutic option for the management of refractory epilepsy in some patients with drug‐resistant epilepsy related to tuberous sclerosis complex.

## CONFLICT OF INTERESTS

Author David E. Burdette has received support from NeuroPace and has served as a paid consultant for NeuroPace, Inc Author Emily A Mirro has equity ownership/stock options with NeuroPace and is an employee of NeuroPace. Author Felicia MK Elefant has equity ownership/stock options with NeuroPace and is an employee of NeuroPace. Author Sanjay E. Patra has received support from and has served as a paid consultant for NeuroPace, Inc. The remaining authors have no conflicts of interest. We confirm that we have read the Journal's position on issues involved in ethical publication and affirm that this report is consistent with those guidelines.
